# Changing trends of the disease burden of non-rheumatic valvular heart disease in China from 1990 to 2019 and its predictions: Findings from global burden of disease study

**DOI:** 10.3389/fcvm.2022.912661

**Published:** 2023-01-19

**Authors:** Xiaoxin Zheng, Qing Guan, Xiuquan Lin

**Affiliations:** ^1^Department of Cardiology, Shengli Clinical Medical College of Fujian Medical University, Fujian Provincial Hospital, Fuzhou, Fujian, China; ^2^Fujian Provincial Key Laboratory of Cardiovascular Disease, Fujian Cardiovascular Institute, Fujian Provincial Center for Geriatrics, Fujian Clinical Medical Research Center for Cardiovascular Diseases, Fuzhou, Fujian, China; ^3^Fujian Heart Failure Center Alliance, Fuzhou, Fujian, China; ^4^Department for Chronic and Non-Communicable Disease Control and Prevention, Fujian Provincial Center for Disease Control and Prevention, Fuzhou, Fujian, China; ^5^The School of Public Health, Fujian Medical University, Fuzhou, Fujian, China

**Keywords:** disease burden, non-rheumatic valvular heart disease, temporal trend, risk factor, prediction

## Abstract

**Objective:**

China has an increasing burden of non-rheumatic valvular heart disease (NRVHD) as the aging of the population is deepening. The aim was to assess the age and sex-specific prevalence and DALYs of NRVHD in China from 1990 to 2019 and to predict the burden in the next 25 years.

**Methods:**

The Global Burden of Disease Study (2019) was used to extract the data of age- and sex-specific incidence, mortality, and disability-adjusted life years (DALYs) of NRVHD in China, 1990-2019. We estimated the annual percentage change (EAPC) to access the temporal trends of the disease burden of NRVHD. The R package called Nordpred was used to perform an age-period-cohort analysis to predict the prevalence of NRVHD in the next 25 years.

**Results:**

The number of incident cases of NRVHD increased from 93.16 thousand in 1990 to 325.05 thousand in 2019. Overall upward trends were observed in the age-standardized incidence rate (ASIR) from 1990 to 2019. Significant temporal trends in mortality and DALYs of NRVHD were observed. High systolic blood pressure, high sodium diet, and lead exposure were the main driving forces for NRVHD. In the next 25 years, the number of new cases and deaths of NRVHD should continue to increase to 390.64 thousand and 10.0 thousand, respectively. The ASIR should show an upward trend, while the ASMR should show a downward trend among men and women.

**Conclusion:**

In China, the overall rates of NRVHD have increased over the past 30 years, and there has been a substantial increase in the burden of NRVHD due to population growth and aging and will continue to increase in the next 25 years. Our results can help shape a multifactorial approach and public policy to reduce the NRVHD burden throughout China.

## 1. Introduction

Valvular heart disease (VHD) is one of the most common heart diseases in China (1). VHD was typically caused by rheumatic heart disease (RHD) previously (2). However, with the rapid development of the economy and the deepening of the aging level in the past 40 years, the incidence of RHD has been significantly reduced while the incidence of non-rheumatic valvular heart disease (NRVHD) is on the rise, especially among the elderly, in China. An inpatient population-based study from a single cardiovascular center in southern China showed that the prevalence of congenital VHD, ischemic VHD, and diabetes heart disease (DHD) is 13.9%, 12.7%, 11.5% respectively, and are markedly increasing especially the prevalence of the DHD (3). Most of the research on heart valve disease prevalence is not to screen all participants with echocardiography, but to make a suspicion based on clinical symptoms followed by echocardiographic confirmation, the prevalence of valvular heart disease obtained by systematic screening with echocardiography is 10 times higher than that of by clinical screening (4), so the results of the current prevalence of NRVHD might be underestimated, and with the projected shift to an older and larger population, the burden of NRVHD will probably increase substantially in the future (1), which highlight the urgency of preventing and treating NRVHD.

Non-rheumatic valvular heart disease is a multifactorial disease caused by both environmental and genetic factors. Some of these factors are not modifiable, such as gender, age, and family history, while others are potentially modifiable, such as hypertension, high salt diet, and lead exposure. These potentially modifiable factors make it possible to control and prevent the incidence of NRVHD. Hypertension and high salt diet are the strongest risk factors for morbidity and mortality from NRVHD. Lifetime exposure to higher systolic blood pressure substantially and casually increased the risk of major valvular heart disease, each genetically-determined 20 mmHg increment in systolic BP almost tripled the odds ratio (OR) of composite valvular heart disease (OR 2.85) (5). High salt intake is closely related to the risks of Cardiovascular disease (6).

The latest Global Burden of Diseases (GBD) study (2019) provides new epidemiological data on the incidence, mortality, and disability-adjusted life-years (DALYs) of NRVHD from 1990 to 2019, enabling us to provide updated estimates of the prevalence and risk factors for NRVHD in China. However, to our knowledge, no recent report has estimated the age-specific and sex-specific prevalence of NRVHD nor has data collected for the GBD study been used to assess NRVHD disability and mortality trends in detail in China (7, 8). Therefore, we described the first comprehensive and systematic assessment of the trends in incidence, deaths, and DALYs from NRVHD by age and sex; investigate major risk factors for NRVHD, and also predicted the numbers and rates of NRVHD incidence and mortality in the next 25 years in China. Our specific research results provided accurate and up-to-date epidemiological information, which is imperative for updating the burden of disease estimates and guiding public health policy decisions making.

## 2. Materials and methods

### 2.1. Data sources

The data on incidence, mortality, and DALYs of NRVHD were downloaded from the website of the Institute for Health Metrics and Evaluation (IHME)^1^, the rules of this selecting data were as follows: location name was “China,” the cause was “non-rheumatic valvular heart disease,” and measures were “incidence,” “mortality,” and “DALYs.” These indicators were calculated with 95% uncertainty intervals (95%UIs).

The World Health Organization (WHO) World Standard Population Distribution (2000-2025) was used as the Standard Population. The date from United Nations World Population Prospects 2019 Revision^2^ was used as the prediction population.

This study has been approved by the Ethics Committee of Wuhan University and Renmin Hospital of Wuhan University. GBD 2019 is a publicly available database without participants’ privacy information.

### 2.2. Evaluation of NRVHD burden

Incidence, mortality, and DALYs with 95% uncertainty intervals (UIs) were estimated using standard methods of the GBD 2019. Estimates of the incidence and prevalence of NRVHD were calculated with the DisMod-MR2.1 (disease-model-Bayesian meta-regression) modeling tool. DisMOd-MR is a Bayesian geospatial disease modeling software that uses various disease parameters, the epidemiological relationships between these parameters, and geospatial relationships to estimate incidence and prevalence. All available high-quality data on incidence, prevalence, and mortality were used to estimate the non-fatal NRVHD burden. All-cause and cause-specific mortality for NRVHD were estimated using the cause of death ensemble modeling. DALYs was the sum of Years lived with disabilities (YLDs) and Years of life lost (YLLs). YLDs were calculated by multiplying the prevalence with the corresponding disability weights. YLLs were calculated by multiplying observed deaths for a specific age by global age-specific reference life expectancy. 95% UIs capturing both random and systematic error in statistical modeling were calculated for all estimates. For the risk factors, the comparative risk assessment (CRA) framework was used to estimate the proportion of DALYs attributable to three well-established risk factors for NRVHD by age and sex: high fasting glucose, high systolic blood pressure, and smoking. The detailed study methods of GBD 2019 have been reported in previous studies (9, 10).

### 2.3. Statistical analysis

The incidence, mortality, and DALYs of NRVHD were performed by age group, sex, and year. The temporal trends for these indicators from 1990 to 2019 were plotted. The age was divided into 18 age groups at 5 years, and 0-39 years were combined into one age group. The time trends of age-standardized incidence (ASIR), age-standardized mortality (ASMR), and age-standardized DALYs rates were described by the estimated annual percentage change (EAPC) which was calculated from a regression model with the natural logarithm of the rate, that is, ln(rate) = α + β × (calendar year) + ε. EAPC was defined as 100 × (exp(β) –1); Its 95% confidence interval (95% CI) was also generated from the fitted model.

We used the power5 APC model of the R package called Nordpred to predict the number and rate of NRVHD incidence and mortality in the next 25 years. Moreover, we estimated the number and rates of NRVHD events by assuming that the events for NRVHD remained stable, decreased, and increased by 1% per year based on the observed data of NRVHD in 2019, in order to facilitate comparison with predicted results. We used the ggplot2packages from the R program (Version 4.1.2; R core team, R Foundation for Statistical Computing, Vienna, Austria) to perform the visualization of the results.

## 3. Results

### 3.1. Incidence, mortality, and DALYs of NRVHD in 2019

In 2019, the number of incident cases and ASIR of NRVHD were 325.05 thousand (95% UI: 307.16, 342.33) and 15.30 per 100,000 (95% UI: 14.51, 16.07) among the total Chinese population, respectively (Table 1). NRVHD contributed to 5.32 thousand (95% UI: 4.51, 6.52) deaths in 2019, and the total population of ASMR was 0.30 per 100,000 (95% UI: 0.26, 0.37) (Table 2). NRVHD caused 170.04 thousand (95% UI: 139.38, 212.06) DALYs in 2019, and the age-standardized rate of DALYs was 9.07 per 100,000 (95% UI: 7.47, 11.17) (Table 3). The number and age-standardized rates of incidence, mortality, and DALYs are about the same for men and women (Tables 1–3).

**TABLE 1:** Number of incident cases and incidence rate of non-rheumatic valvular heart disease in China in 1990 and 2019 and EAPC from 1990 to 2019.

Characteristics	1990		2019		1990-2019
	**Incident cases** **[× 10^3^** **(95% UI)]**	**Incident rate** **[per 100,000** **(95% UI)]**	**Incident cases** **[× 10^3^** **(95% UI)]**	**Incident rate** **[per 100,000** **(95% UI)]**	**EAPC in incidence rate** **[%(95% CI)]**
Overall*	93.16 (88.80, 98.01)	9.22 (8.76, 9.69)	325.05 (307.16, 342.33)	15.30 (14.51, 16.07)	3.63 (0.33, 7.05)
Sex*					
Male	40.21 (38.12, 42.36)	7.75 (7.34, 8.15)	140.35 (131.63, 148.75)	13.25 (12.47, 13.97)	3.55 (0.58, 6.61)
Female	52.95 (50.39, 55.60)	10.81 (10.28, 11.35)	184.7 (175.14, 194.42)	17.37 (16.49, 18.21)	3.73 (0.09, 7.50)
Age at diagnosis**(year)					
0-14	0	0	0	0	—
15-19	0.81 (0.74, 0.89)	0.64 (0.58, 0.70)	0.53 (0.48, 0.58)	0.70 (0.64, 0.77)	3.54 (–0.99, 8.28)
20-24	2.60 (2.37, 2.86)	1.96 (1.79, 2.16)	1.92 (1.76, 2.10)	2.34 (2.15, 2.56)	3.91 (–0.67, 8.70)
25-29	3.78 (3.47, 4.16)	3.43 (3.15, 3.77)	4.44 (4.07, 4.85)	4.01 (3.68, 4.38)	4.07 (–0.51, 8.85)
30-34	4.42 (4.06, 4.84)	4.99 (4.59, 5.47)	7.47 (6.86, 8.16)	5.79 (5.31, 6.32)	3.98 (–0.58, 8.75)
35-39	5.59 (5.12, 6.14)	6.10 (5.59, 6.71)	7.76 (7.13, 8.47)	7.69 (7.07, 8.40)	3.81 (–0.72, 8.56)
40-44	6.78 (5.89, 7.76)	10.09 (8.76, 11.54)	13.86 (12.2, 15.65)	13.63 (12.01, 15.40)	3.66 (–0.20, 7.67)
45-49	8.74 (6.64, 11.17)	16.89 (12.84, 21.60)	28.76 (23.22, 35.24)	23.69 (19.13, 29.03)	3.78 (0.25, 7.43)
50-54	13.69 (12.01, 15.55)	28.63 (25.13, 32.53)	57.13 (51.50, 63.10)	45.67 (41.16, 50.44)	4.29 (0.71, 7.99)
55-59	19.46 (16.35, 22.64)	44.76 (37.62, 52.08)	79.80 (69.93, 90.27)	84.15 (73.74, 95.19)	4.58 (0.88, 8.41)
60-64	15.37 (13.11, 17.83)	43.38 (37.00, 50.34)	66.68 (59.42, 74.32)	84.88 (75.64, 94.61)	4.13 (0.66, 7.71)
65-69	7.22 (5.65, 9.21)	26.38 (20.64, 33.64)	34.47 (28.36, 41.92)	48.97 (40.29, 59.56)	3.42 (0.60, 6.33)
70-74	2.91 (1.68, 4.50)	15.46 (8.89, 23.86)	13.15 (8.41, 18.58)	27.48 (17.56, 38.82)	2.95 (0.71, 5.23)
75-79	1.18 (0.75, 1.65)	10.32 (6.56, 14.49)	5.17 (3.60, 6.93)	17.33 (12.05, 23.21)	2.55 (0.63, 4.50)
80-84	0.44 (0.23, 0.70)	7.76 (4.08, 12.33)	2.40 (1.44, 3.63)	12.58 (7.55, 19.06)	2.15 (0.66, 3.66)
85-89	0.15 (0.09, 0.23)	7.98 (4.84, 11.95)	1.11 (0.67, 1.68)	13.08 (7.82, 19.71)	2.09 (0.84, 3.36)
90-94	0.03 (0.02, 0.05)	8.63 (5.49, 12.93)	0.33 (0.19, 0.50)	14.63 (8.54, 22.27)	1.96 (1.00, 2.92)
95 +	0.01 (0, 00.010)	9.93 (5.49, 16.38)	0.08 (0.03, 0.15)	17.47 (7.55, 33.79)	1.87 (0.92, 2.83)

EAPC, estimated annual percentage change; 95% UI, 95% uncertainty interval; 95% CI, 95% confidence interval; *, age-standardized incidence rate; **, the crude incidence rate in each age group.

**TABLE 2:** Number of deaths and mortality rate of non-rheumatic valvular heart disease in China in 1990 and 2019 and EAPC from 1990 to 2019.

Characteristics	1990		2019		1990-2019
	**Deaths cases** **[× 10^3^** **(95% UI)]**	**Mortality rate** **[per 100,000** **(95% UI)]**	**Deaths cases** **[× 10^3^** **(95% UI)]**	**Mortality rate** **[per 100,000** **(95% UI)]**	**EAPC in mortality rate** **[%(95% CI)]**
Overall*	3.75 (2.88, 4.57)	0.56 (0.43, 0.67)	5.32 (4.51, 6.52)	0.30 (0.26, 0.37)	−2.51 (–3.56, –1.45)
Sex*					
Male	1.86 (1.41, 2.43)	0.58 (0.45, 0.79)	2.96 (2.31, 3.90)	0.37 (0.30, 0.50)	−1.89 (−3.00, −0.76)
Female	1.89 (1.23, 2.43)	0.53 (0.36, 0.68)	2.36 (1.89, 2.92)	0.25 (0.20, 0.30)	−3.02 (−4.04, −1.99)
Age at diagnosis**(year)					
0-14	0		0		—
15-19	0.06 (0.05, 0.09)	0.05 (0.04, 0.07)	0.02 (0.01, 0.02)	0.02 (0.02, 0.03)	−3.89 (−5.07, −2.69)
20-24	0.08 (0.06, 0.11)	0.06 (0.04, 0.09)	0.03 (0.02, 0.03)	0.03 (0.03, 0.04)	−3.48 (−4.74, −2.20)
25-29	0.09 (0.06, 0.12)	0.08 (0.05, 0.11)	0.05 (0.04, 0.06)	0.05 (0.04, 0.06)	−2.88 (−4.27, −1.47)
30-34	0.09 (0.06, 0.12)	0.10 (0.07, 0.14)	0.08 (0.07, 0.10)	0.06 (0.05, 0.07)	−2.81 (−4.34, −1.25)
35-39	0.14 (0.10, 0.18)	0.15 (0.11, 0.20)	0.09 (0.08, 0.11)	0.09 (0.08, 0.11)	−3.01 (−4.69, −1.32)
40-44	0.15 (0.11, 0.19)	0.22 (0.16, 0.28)	0.13 (0.10, 0.16)	0.13 (0.10, 0.16)	−2.97 (−4.54, −1.38)
45-49	0.14 (0.10, 0.19)	0.28 (0.20, 0.37)	0.19 (0.15, 0.23)	0.15 (0.12, 0.19)	−2.99 (−4.57, −1.39)
50-54	0.21 (0.15, 0.28)	0.45 (0.32, 0.59)	0.30 (0.24, 0.38)	0.24 (0.19, 0.31)	−2.99 (−4.46, −1.49)
55-59	0.30 (0.22, 0.38)	0.68 (0.51, 0.87)	0.34 (0.28, 0.43)	0.36 (0.29, 0.45)	−2.85 (−4.25, −1.44)
60-64	0.36 (0.27, 0.45)	1.02 (0.76, 1.28)	0.45 (0.37, 0.55)	0.58 (0.47, 0.71)	−2.80 (−4.81, −0.74)
65-69	0.45 (0.34, 0.57)	1.65 (1.23, 2.07)	0.71 (0.59, 0.86)	1.00 (0.83, 1.22)	−2.51 (−4.51, −0.48)
70-74	0.47 (0.35, 0.57)	2.48 (1.87, 3.02)	0.68 (0.57, 0.83)	1.43 (1.20, 1.74)	−2.31 (−3.40, −1.21)
75-79	0.46 (0.36, 0.56)	4.05 (3.16, 4.87)	0.71 (0.60, 0.85)	2.37 (2.01, 2.86)	−2.07 (−3.12, −1.01)
80-84	0.42 (0.33, 0.53)	7.48 (5.93, 9.31)	0.73 (0.62, 0.93)	3.85 (3.24, 4.86)	−2.39 (−3.62, −1.14)
85-89	0.24 (0.19, 0.30)	12.29 (9.79, 15.70)	0.53 (0.44, 0.69)	6.20 (5.22, 8.16)	−2.40 (−3.76, −1.02)
90-94	0.08 (0.06, 0.10)	21.12 (16.04, 28.00)	0.21 (0.17, 0.26)	9.38 (7.53, 11.38)	−3.12 (−4.43, −1.80)
95 +	0.02 (0.01, 0.02)	27.49 (20.56, 36.51)	0.07 (0.05, 0.08)	15.27 (11.53, 18.80)	−2.65 (−3.94, −1.34)

EAPC, estimated annual percentage change; 95% UI, 95% uncertainty interval; 95% CI, 95% confidence interval; *, age-standardized mortality rate; **, the crude mortality rate in each age group.

**TABLE 3:** Number of DALYs and DALYs rate of non-rheumatic valvular heart disease in China in 1990 and 2019 and EAPC from 1990 to 2019.

Characteristics	1990		2019		1990-2019
	**DALYs** **[× 10^3^** **(95% UI)]**	**DALYs rate** **[per 100,000** **(95% UI)]**	**DALYs** **[× 10^3^** **(95% UI)]**	**DALYs rate** **[per 100,000** **(95% UI)]**	**EAPC in DALYs rate** **[%(95% CI)]**
Overall*	116.39 (89.54, 143.63)	13.43 (10.50, 16.22)	170.04 (139.38, 212.06)	9.07 (7.47, 11.17)	−1.83 (−3.02, −0.62)
Sex*					
Male	60.57 (46.78, 79.32)	13.92 (10.8, 17.99)	91.95 (72.55, 117.39)	10.26 (8.15, 13)	−1.49 (−2.73, −0.24)
Female	55.81 (36.95, 72.68)	12.82 (8.84, 16.36)	78.09 (61.09, 99.97)	7.92 (6.21, 10.14)	−2.17 (−3.38, −0.94)
Age at diagnosis**(year)					
0-14	0	0	0	0	—
15-19	4.59 (3.26, 6.23)	3.62 (2.57, 4.91)	1.14 (0.95, 1.41)	1.52 (1.26, 1.88)	−3.86 (−5.03, −2.67)
20-24	5.52 (3.83, 7.66)	4.17 (2.89, 5.78)	1.71 (1.40, 2.09)	2.09 (1.71, 2.56)	−3.45 (−4.71, −2.18)
25-29	5.38 (3.59, 7.62)	4.88 (3.26, 6.91)	3.26 (2.70, 3.90)	2.94 (2.44, 3.52)	−2.85 (−4.23, −1.45)
30-34	5.04 (3.57, 6.94)	5.69 (4.03, 7.84)	4.64 (3.88, 5.54)	3.59 (3.00, 4.29)	−2.76 (−4.29, −1.22)
35-39	7.09 (5.14, 9.52)	7.75 (5.62, 10.40)	4.87 (4.02, 5.87)	4.83 (3.98, 5.82)	−2.94 (−4.61, −1.26)
40-44	6.97 (5.20, 9.03)	10.37 (7.73, 13.43)	6.29 (5.08, 7.68)	6.19 (5.00, 7.55)	−2.89 (−4.44, −1.30)
45-49	6.33 (4.57, 8.25)	12.24 (8.84, 15.96)	8.44 (6.76, 10.24)	6.95 (5.57, 8.43)	−2.81 (−4.38, −1.23)
50-54	8.57 (6.41, 11.17)	17.94 (13.4, 23.36)	12.87 (10.25, 15.89)	10.29 (8.19, 12.7)	−2.66 (−4.09, −1.20)
55-59	10.89 (8.31, 13.68)	25.05 (19.13, 31.47)	14.35 (11.34, 18.27)	15.13 (11.96, 19.26)	−2.29 (−3.68, −0.89)
60-64	12.01 (9.31, 14.89)	33.89 (26.29, 42.05)	18.32 (14.41, 23.71)	23.32 (18.34, 30.18)	−2.07 (−4.22, 0.13)
65-69	13.09 (10.10, 16.4)	47.85 (36.90, 59.92)	25.12 (20.13, 32.41)	35.69 (28.60, 46.05)	−1.74 (−3.91, 0.47)
70-74	11.54 (9.04, 14.34)	61.20 (47.94, 76.06)	21.90 (17.56, 28.16)	45.75 (36.7, 58.85)	−1.37 (−2.51, −0.20)
75-79	9.30 (7.34, 11.35)	81.53 (64.35, 99.50)	18.55 (14.95, 24.01)	62.16 (50.08, 80.45)	−1.08 (−2.24, 0.10)
80-84	6.45 (5.20, 7.92)	114.46 (92.15, 140.51)	15.68 (12.31, 20.39)	82.21 (64.56, 106.91)	−1.16 (−2.48, 0.18)
85-89	2.77 (2.22, 3.48)	144.60 (116.02, 181.44)	9.03 (7.09, 11.86)	106.21 (83.40, 139.51)	−1.04 (−2.48, 0.41)
90-94	0.70 (0.55, 0.90)	189.01 (147.88, 241.40)	3.00 (2.31, 3.92)	133.72 (102.92, 174.79)	−1.47 (−2.94, 0.01)
95 +	0.13 (0.10, 0.16)	210.03 (162.80, 262.95)	0.87 (0.66, 1.11)	195.82 (148.25, 248.11)	−0.74 (−2.29, 0.83)

DALYs, disability-adjusted life-years; EAPC, estimated annual percentage change; 95% UI, 95% uncertainty interval; 95% CI, 95% confidence interval; *, age-standardized DALYs rate; **, crude DALYs rate in each age group.

**FIGURE 1:**
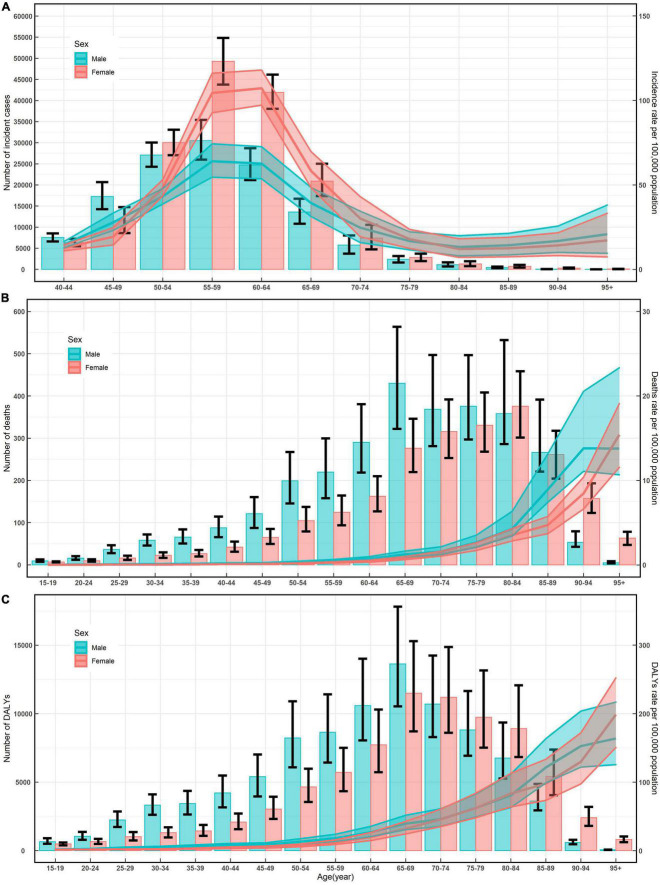
Numbers and rates of incidence **(A)**, death **(B)**, and DALYs **(C)** of non-rheumatic valvular heart disease by age and sex in 2019 in China. Shading represents the upper and lower limits of the 95% uncertainty intervals (95% UIs). DALYs, disability-adjusted life-years.

In 2019, the number of incident cases of NRVHD reached a peak among the total population aged 55-59 years (Table 1), and this trend was similar for men and women (Figure 1A). The number of deaths of NRVHD reached a peak among the total population aged 80-84 years (Table 2), which trend did not differ in women, while the number of deaths in men peaked at 65-69 years old (Figure 1B). The number of DALYs reached a peak at 65-69 years old among the total population and both sexes (Table 3 and Figure 1C). The number of incident cases in women was significantly higher than that in men between the ages of 50-74 years old, while the differences in incident cases were not significant at other ages in both men and women (Figure 1A). The numbers of deaths and DALYs were higher among men than women in individuals in most age-specific groups (Figures 1B, C).

The age-specific rates for incidence reached a peak among the total population aged 60-64 years (Table 1); the age-specific rates for mortality and DALYs increased with increasing age (Tables 2, 3). The trends of age-specific rates of incidence, mortality, and DALYs among both men and women were similar to the trends for the total population. Furthermore, the numbers and rates of incidence were concentrated in the population aged 50-89 years old, while the numbers and rates of deaths and DALYs were concentrated in the elderly population (≥ 60 years old) (Figure 1).

### 3.2. Temporal trends of incidence, mortality, and DALYs of NRVHD from 1990 to 2019

**FIGURE 2:**
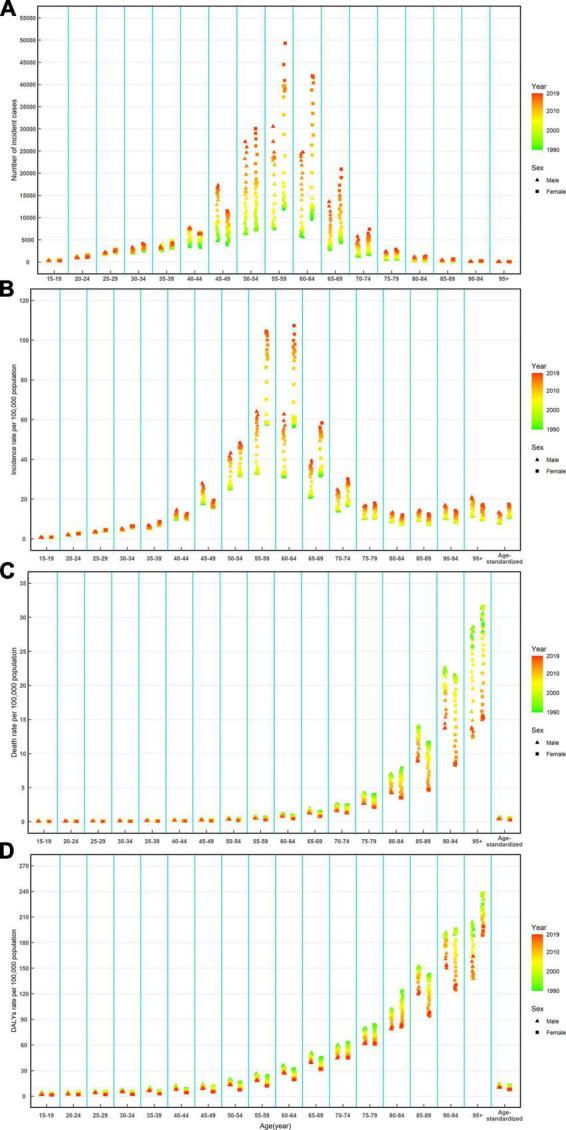
Number of incident cases **(A)**, incidence rate **(B)**, death rate **(C)**, and DALYs rate **(D)** of Non-rheumatic valvular heart by age and sex, from 1990 to 2019 in China. DALYs, disability-adjusted life-years.

From 1990 to 2019, the number of incident cases, deaths, and DALYs of NRVHD all significantly increased among the total population (Tables 1–3). The number of incident cases increased by more than two times among men ≥ 45 years old and women ≥ 50 years old during the study period (Figure 2A). The ASIR was 9.22 per 100,000 (95%UI: 8.76, 9.69) in 1990, which increased in 2019, with an EAPC of 3.63 (95%CI: 0.33, 7.05) in the total population (Table 1). The ASIR of women increased more significantly than that of men during this period [EAPC = 3.73, 95%CI: (0.09, 7.50) *vs* EAPC = 3.55, 95%CI: (0.58, 6.61), respectively] (Table 1). Additionally, overall upward trends in the incidence rates were observed among both sexes in all age-specific groups (Figure 2B).

The ASMR decreased from 1990 [0.56 per 100,000 (95%UI: 0.43, 0.67)] to 2019, with an EAPC of −2.51 (95%CI: −3.56, −1.45) (Table 2). A decreasing trend of age-standardized DALYs was also observed during this period, and the EAPC was −1.83 (95%CI: −3.02, −0.62) (Table 3). Overall downward trends in mortality and DALYs rates were observed in most age-specific groups and both sexes from 1990 to 2019 (Figures 2C, D).

### 3.3. Mortality and DALYs rates of NRVHD attributable to risk factors and their temporal trends from 1990 to 2019

**FIGURE 3:**
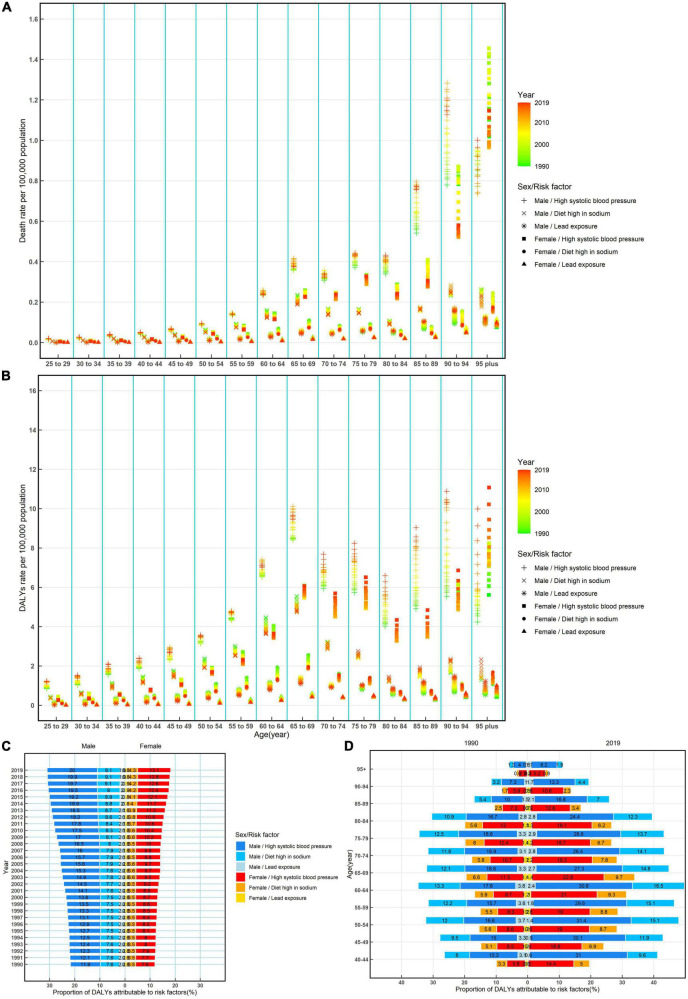
Rates of death, rates, and proportions of DALYs attributable to risk factors by age and sex, from 1990 to 2019 in China. Rates of death **(A)** and DALYs **(B)** of non-rheumatic valvular heart attributable to risk factors by age and sex, from 1990 to 2019 in China; proportions of DALYs attributable to risk factors by sex from 1990 to 2019 in China **(C)**; and proportions of DALYs attributable to risk factors by age and sex in 1990 and 2019 in China **(D)**. DALYs, disability-adjusted life-years.

In most age-specific groups, the mortality that was attributed to high systolic blood pressure was the highest among men and that was attributed to lead exposure was the lowest among women (Figure 3A). The overall downward trends attributable to a high-sodium diet were observed in most age-specific groups and both sexes, trends of NRVHD mortality attributed to high systolic blood pressure decreased in women while increased in men in most age-specific groups, trends of NRVHD mortality attributed to lead exposure was decreased under the age of 70 while was increased over the age of 70 among men and women (Figure 3A). Moreover, the temporal trends of the rates of DALYs attributable to a high-sodium diet, high systolic blood pressure, and lead exposure were similar to that of mortality (Figure 3B).

The proportions of DALYs that were attributed to risk factors (high systolic blood pressure, a high-sodium diet, and lead exposure) were different between men and women. High systolic blood pressure was the most significant contribution among both sexes, accounting for more than 11.9% of DALYs in men and accounting for more than 7.6% of DALYs in women, and the proportions of DALYs attributed to high systolic blood pressure increased over time from 1990 to 2019 (Figure 3C).

Moreover, during this period, the proportions of DALYs attributable to high systolic blood pressure, a high-sodium diet, and lead exposure show a notable difference between sexes in most age-specific groups, the proportions of DALYs attributable to three risk factors were higher in men than in women in individuals. During this period, the proportions of DALYs attributable to high systolic blood pressure, a high-sodium diet, and lead exposure were observed to have a relatively flat increase, but then a drastic decrease in >65 years old among men and women (Figure 3D).

### 3.4. Predictions of incidence and mortality of NRVHD from 2020 to2044

**FIGURE 4:**
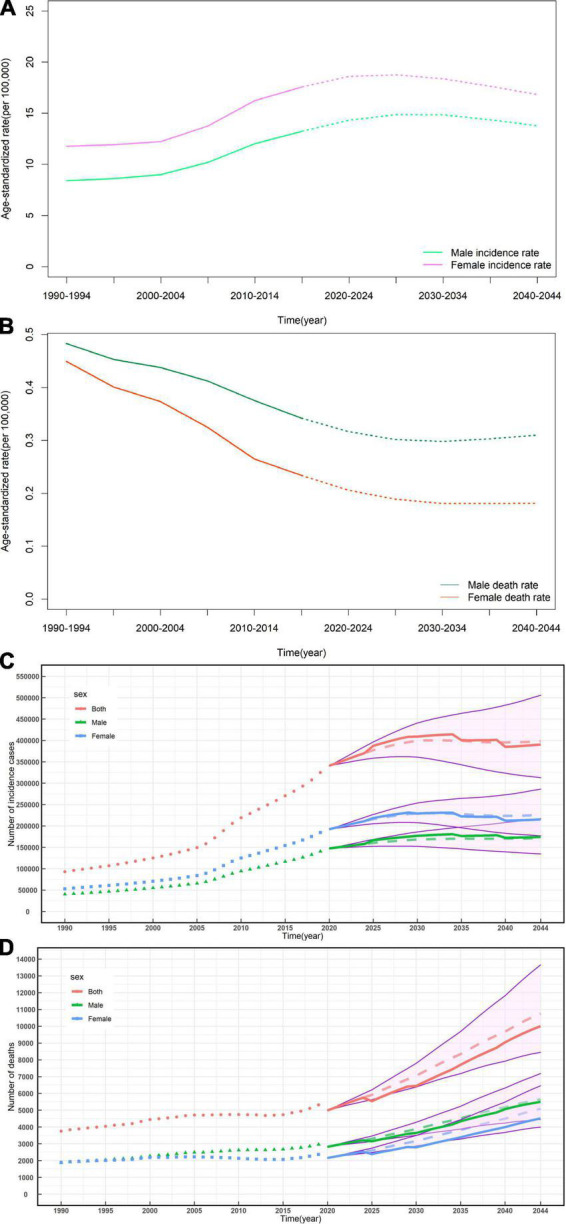
Temporal trends and forecasted rates of incidence **(A)** and death **(B)**, and the number of incident cases **(C)** and deaths **(D)** of non-rheumatic valvular heart by sex, from 2020 to 2044 in China. Solid lines and dash lines represent the observed and the predicted number of incident cases and deaths of non-rheumatic valvular heart; shading represents a 1% decrease and increase interval based on the 2019 rate. DALYs, disability-adjusted life-years.

Based on GBD data of NRVHD from 1990 to 2019 in China, we further predicted the numbers and rates of incidence and mortality in the next 25 years (Figure 4). In the next 25 years, notable differences between rates of incidence and mortality, the incidence rates should be increasing while the death rate should be decreasing among both men and women (Figures 4A, B), while the numbers of new cases and deaths of NRVHD should continue to increase consistently from 2020 to 2044 (Figures 4C, D). In 2044, the overall number of new NRVHD cases should increase to 390.64 thousand (Figure 4C) and the number of NRVHD deaths should increase to 10.0 thousand (Figure 4D). In 2044, among men, the number of incident cases and deaths should increase to 174.32 thousand and 5.51 thousand, respectively (Figures 4C, D). Among women, the number of incident cases and deaths should increase to 215.47 thousand and 4.51 thousand in 2044, respectively (Figures 4C, D).

## 4. Discussion

Non-rheumatic valvular heart disease (NRVHD) is a global health issue and has led to a high burden of VHD in elderly people worldwide (11–13). China is one of the countries with the highest burden of NRVHD in the world. Few studies analyzed the burden of NRVHD in China in the past, and the latest disease burden of NRVHD is still unclear (14). New epidemiological data of the Global Burden of Disease (GBD) 2019 study on NRVHD at macro- and meso-level geographic scales (8), enables us to provide the most consistent, transparent, and up-to-date overview of the prevalence and risk factors for NRVHD nationally, for the first time. In this investigation, we assessed the burden of NRVHD comprehensively, its temporal trends by sex, age, and risk factors in depth and uniquely, and predicted the burden in the next 25 years in China. Our study has shown that there has been an increasing trend in the incidence of NRVHD among the total Chinese population from 1990 to 2019. High systolic blood pressure, diet high-sodium, and lead exposure were confirmed to be associated with a higher risk of NRVHD. With the projected shift to an older and larger population (15), the burden of NRVHD will probably increase substantially in the future. This is consistent with the results predicted by our prediction model. For the next 25 years, it demonstrates that the number of new cases and deaths of NRVHD should keep on increasing among both sexes. Meanwhile, the incidence rate showed an overall increasing trend, while the death rate revealed an overall decreasing trend. Despite clinical practice guidance and current knowledge to reduce the burden of NRVHD morbidity, it is clear that the continued heavy burden of NRVHD in China may be shifting to mortality from chronic morbidity.

Our study indicates that the age-standardized prevalence and DALYs rates of NRVHD are prevalent in China based on the data from GBD 2019, which is similar to the level worldwide. Although the age-standardized prevalence and DALYs rates are stable among the Chinese population basically, the significant increase in NRVHD cases and DALYs in China draws more attention. The increased prevalence of may attribute to the following reasons. First, recent studies have shown that the elderly population (individuals aged ≥ 60 years) is estimated to increase to 300 million by the end of 2025. It may cause the heavy disease burden of NRVHD in the elderly population, which is consistent with our results. Meanwhile, China has the largest population base in the world, leading to higher new cases and rates of incidence, mortality, and DALYs. Second, some projects were launched by the Chinese government for the populations with a high risk of NRVHD. In 2015, the National Cardiovascular Disease Center systematically and continuously carried out cardiovascular disease prevention and control skills training for grass-roots medical personnel through the implementation of the “grass-roots cardiovascular disease comprehensive risk management project”. Thus, significant improvement has been made aiming at early diagnosis and treatment of NRVHD among both sexes, leading to an overall decline in the rates of death of NRVHD, and on the other hand, newly detected cases would increase, keeping the rates of incidence and DALYs from declining notably. Thirdly, increased lifestyle, cardiovascular care, and metabolic risk factors are also major contributors among younger populations. With the improvement of the changing lifestyles and public health in China, the risk factors, such as high systolic blood pressure, a diet high in sodium, and lead exposure, have been effectively controlled, leading to a decline in the rates of mortality.

The observed NRVHD prevalence in our study suggests increased exposure to NRVHD risk factors including high systolic blood pressure, a diet high in sodium, lead exposure, and high fasting plasma glucose, which is consistent with former data (16–20), implying the importance of proper control of these risk factors. It showed that 23.2% of Chinese people aged 18 or older had hypertension (21). High blood pressure in patients with hypertension brings extra mechanical pressure to the valve. Over time, the collagen fibers in the valve wear and break, resulting in calcium salt deposition in the crack, leading to calcified lesions of the valve. As for lead exposure, it was explained by evidence that chronic exposure to lead is a factor resulting in hypertension and atherosclerosis (18, 22–24). Overall, lead exposure can reduce nitric oxide availability, promote vascular inflammation *via* oxidative stress, and increase the production of endothelin, resulting in high blood pressure, which may be explained by evidence that chronic exposure to lead is a factor contributing to hypertension and calcified lesions of the valve (22–26). From our study, we found that the proportion of DALYs attributable to high systolic blood pressure and lead exposure was more than 13.9% in China in 2019 among men and women. In China, healthy China 2030 has pushed forward the prevention and treatment of NRVHD with or without other cardiovascular diseases, promoted the research and formulation of the technical scheme and enforcement path of “three highs” (hypertension, hyperlipidemia, and hyperglycemia). Additionally, the Work Safety Law of the People’s Republic of China (PRC) and the Law of the PRC on Prevention and Control of Occupational Diseases officially modified the occupational health regulatory system in lead exposure control, which is of great significance to reduce the burden of NRVHD (27, 28). The benefits of protection from lead exposure in reducing the risk of NRVHD have also been supported in our data. Trends of NRVHD mortality attributed to lead exposure decreased under the age of 70 among both sexes from 1990 to 2019.

Our sex-specific and age-specific prevalence data models have improved the precision of our estimation regarding another risk factor of a diet high in sodium. Potential explanations for the association between a diet high in sodium and aortic valve disease include that diets high in sodium may be associated with the effect of aldosterone on renal mineralocorticoid receptors (29, 30), which leading to CAVD (31). Furthermore, studies have suggested that a high sodium intake leads to the occurrence of high blood pressure (32–34), which is another possible explanation for the relationship between a diet high in sodium and CAVD (32, 34). Our study demonstrated that the proportion of DALYs attributable to a diet high in sodium was more than 4.3% in China in 2019 among both sexes. To date, salt intake in China is high with a daily average intake of 12–14 g (35). By 2030, the national per capita daily salt intake will be reduced by 20% (36). The above measures may be beneficial to decrease in the prevalence and burden of NRVHD.

Although the overall downward trends in mortality and DALYs rates were observed in most age-specific groups and both sexes from 1990 to 2019, the numbers of incident cases, deaths, and DALYs of NRVHD all significantly increased among the total population. The total expenses for CVD hospitalization have increased rapidly since 2004, much faster than the increase in gross domestic product. Moreover, with an aging population and global economic growth, an increasingly large number of NRVHD cases will pose a heavy economic burden on health care and treatment. We further predicted that the numbers of new cases, deaths of NVHD, and incidence rates will continue to increase consistently, while the death rate should be decreasing among both sexes from 2020 to 2044. Multifactorial national epidemiology policies can improve epidemiological trends and explain the decreasing death rate. Healthy China 2030 highlights that it is necessary to guide population to have a reasonable diet. In 2020, China’s guidelines for the primary prevention of cardiovascular diseases proposed that blood pressure management should be carried out to reduce blood pressure and control other risk factors. China’s Medium and Long Term Plan of Preventing and Controlling Chronic Diseases set the goal to reduce CVD mortality rates by 15% by 2025 partly by preventing high risk factors including lead exposure. The national nutrition plan (2017-2030) puts forward the action goal of reducing the national per capita daily salt intake by 20% by 2030. Although an astonishing fact is that NRVHD is generally ignored in China, it highlighted that greater efforts and special attention should be paid to targeted public health strategies.

Our study has several limitations to be acknowledged. First, we only assessed the disease burden of NRVHD at the national level but did not provide some more details provincially. Second, because of the progressive and slow nature of valvular heart diseases, many severe valve lesions remain undiagnosed due to neglected symptoms from the patients (37). Meanwhile, valve diseases are poorly represented in the international diseases classification, and their contribution to morbidity and mortality might have been ignored without echocardiography (1). Thus the trends in the burden of NRVHD may have been underestimated. Third, it has been described previously how the limitations of the GBD methodology affect related studies (7, 38–41). The GBD study cannot capture the most recent changes in health status, because of the time lags in the reporting of health information by government sectors. Fourth, the inclusion of more data on risk factors for NRVHD would lead to a precise understanding of the prevalence of NRVHD and a more scientific preventive strategy. We could not evaluate the NRVHD burden caused by other important risk factors such as high fasting plasma glucose and red meat, because corresponding data is missing in the GBD database. Although the data used to estimate the prevalence in our study were corrected, filled, and fitted through various models, non-determinacy still needs to be considered when interpreting our results.

## 5. Conclusion

A substantially high prevalence of NRVHD was observed in the recent three decades, especially in the elderly. Even larger numbers of new cases and deaths of NRVHD are to be expected. The ASIR and ASMR should show an upward and downward trend among both sexes, respectively. It may lead to high healthcare costs and signify the potential for even higher costs in the next 25 years. The cardiovascular risk factors of high systolic blood pressure, a diet high in sodium, and lead exposure are positively associated with NRVHD in China over this time period. Our study results are valuable in drawing attention to the control and treatment of NRVHD, more targeted strategies for NRVHD control and prevention are needed to reduce negative health outcomes.

## Data availability statement

The datasets presented in this study can be found in online repositories. The names of the repository/repositories and accession number(s) can be found in the article/supplementary material.

## Author contributions

XZ: conceptualization, writing – original draft, and formal analysis. YH: writing – review. XL: conceptualization, methodology, software, and visualization. QG: conceptualization and data curation. All authors contributed to the article and approved the submitted version.

## Footnotes

1
http://ghdx.healthdata.org/gbd-results-tool

2
https://population.un.Org/WPP/Download/Standard/Population/
